# Prediction accuracy of cochlear implant electrode insertion using the OTOPLAN software

**DOI:** 10.1007/s00405-025-09525-3

**Published:** 2025-06-25

**Authors:** Caterina Vazzana, Marten Geisen, Uwe Baumann, Dennis Sakmen, Esther Knörle, Timo Stöver, Silke Helbig

**Affiliations:** 1 Department of Otorhinolaryngology, Head and Neck Surgery, Goethe University Frankfurt, University Hospital, Frankfurt, Germany; 2 Department of Audiology & Audiological Acoustic, Goethe University Frankfurt, University Hospital, Frankfurt, Germany

**Keywords:** Cochlear implant, OTOPLAN, Insertion angle, Electrode carrier, Insertion depth

## Abstract

**Purpose:**

Preoperative cochlear measurements to determine optimal electrode array length are increasingly used in clinical practice. This study aims to evaluate the predictive accuracy of OTOPLAN software for cochlear implant (CI) electrode array insertion depth, angle, and frequency allocation in a clinical setting.

**Methods:**

Data from 121 patients implanted with a CI between 2016 and 2020 with both preoperative CT and postoperative CBCT DICOM data were analysed. Parameters measured included cochlear diameter (A), height (H), width (B), cochlear duct length (CDL), insertion angle, insertion depth and frequency. Statistical significance of differences between CT and CBCT measurements was determined using a p-value threshold of < 0.05.

**Results:**

Data from 121 patients were analysed. Median measurements for CT vs. CBCT were 9.2 mm vs. 9.5 mm for A, 4.2 mm vs. 4.3 mm for H, and 6.8 mm for B in both modalities. The median CDL was 35.9 mm vs. 36.3 mm. Significant differences were observed for parameters A, B and CDL, while no significant difference was found for H. CDL measurements were significantly shorter in females compared to males. In addition, significant discrepancies were found between CT and CBCT for insertion angles, insertion depths and frequency estimates.

**Conclusion:**

OTOPLAN is effective for predicting insertion depth with high accuracy. However, significant differences between CT- and CBCT-derived measurements suggest that CT is the more reliable modality for OTOPLAN-assisted evaluation of cochlear parameters, due to CBCT’s susceptibility to motion artefacts.

## Introduction and background

Anatomical and radiological studies have shown that the size and shape of the cochlea varies from person to person. There can even be differences between the right and left ear of the same person [1–4]. Since the late 1990s, there has been a paradigm shift towards less invasive, partially hearing preserving cochlear implant (CI) surgery [14]. The materials used and the design and length of the electrode array have been adapted accordingly. Preservation of residual hearing - often through the use of shorter electrode arrays or shallower insertion depths - allows patients to benefit from their natural residual hearing [5, 6]. On the other hand, deeper insertion appears to be advantageous for hearing impaired patients with no usable residual hearing or in cases where the loss of residual hearing is foreseeable. Deeper placement in the cochlea, which provides full coverage of the cochlea, directly stimulates the apical neuronal structures of the cochlea. As a result, better speech understanding can be achieved than with a shorter insertion [7].

The choice of the optimal electrode length is an essential aspect of patient counselling prior to cochlear implantation. Preoperative measurement of the cochlea to be implanted plays a central role in this [8–13]. The visualisation and measurement of the cochlea, which at this stage is mainly carried out by radiologists, has become increasingly important [2, 14, 15]. Variables such as diameter, width and height of the cochlea are recorded and approximate formulae have been developed to calculate the length of the cochlear duct. Since 2018, the OTOPLAN software (CAScination, Bern, Switzerland) has been available for this purpose, which automates this calculation using slice image data sets. This software allows the user to systematically measure the cochlea and thus determine the length of the cochlear duct (CDL) [16, 17]. In addition, OTOPLAN provides the ability to simulate the implantation of electrode arrays of different lengths, allowing precise frequency mapping for individual contacts on the electrode array. This information on the expected position of the electrode within the cochlea is clinically relevant and supports the selection of the individual electrode length [18]. It offers the potential for frequency-based fitting of the implant [18].

For preoperative evaluation prior to cochlear implantation, 3D imaging is standard, usually temporal bone computed tomography (CT) to visualise the bony structures and allow side-by-side comparison. For post-operative monitoring, many centres prefer intra- or post-operative cone beam computed tomography (CBCT) of the implanted side, as it has a lower radiation exposure than repeat CT [20, 21].

This retrospective study evaluates the predictive accuracy of OTOPLAN calculations regarding the insertion depth of the electrode carrier in a clinically common setting.

## Materials and methods

### Patients

This retrospective study was reviewed and approved by the local ethics committee (no. 20/608). All patients who received a unilateral or bilateral CI at a university hospital specialising in CIs between 01.01.2016 and 01.04.2020 were included in the analysis. The inclusion criteria were as follows:

1. Implantation was performed with Flex electrodes (MED-EL, Innsbruck, Austria). These straight electrode arrays, designed for insertion along the lateral cochlear wall, have 12 contacts and are available in different lengths. This criterion was chosen because OTOPLAN was specifically designed to simulate MED-EL electrodes.
2. Electrode arrays had to be fully implanted; patients with evidence of incomplete implantation (less than 12 intracochlear contacts) were excluded.
3. Both preoperative CT and postoperative CBCT had to be available; patients who did not meet these requirements, e.g. by using alternative imaging techniques, were excluded.
4. All pre- and post-operative images had to be taken at the university hospital where the study was conducted; external images were not included in the analysis.
5. Only cochleae with normal anatomy were included in the study; malformed cochleae were excluded.

A total of 121 patients were included in the analysis. Of the 121 patients, 18 were either treated bilaterally in one surgery or received a second consecutive CI. A total of 139 CI ears could therefore be analysed. There were 84 female and 55 male ears. The average age of the patients at the time of surgery was 61 years. The youngest patient was 11 years old and the oldest was 88 years old. 139 electrodes were implanted, ranging in length from 20 mm to 28 mm.

The standard CBCT used had a resolution of 0.15 mm, whereas conventional CT typically offers a resolution of between 0.4 and 0.6 mm. This illustrates the higher level of spatial detail that can be achieved with CBCT, particularly with regard to fine bony structures.

### Surgical procedure

The surgical procedure was standardised in all cases and consisted of a mastoid approach followed by a posterior tympanotomy. Electrodes were inserted through the previously exposed round window, the overhang of which was removed. In cases where the diameter of the round window was too small for insertion, the window was enlarged laterally by reducing the hook area. Insertion itself was performed very slowly and according to the surgeon’s judgement, with the aim of inserting the electrode carrier completely up to the marker point.

### Anatomical measurement

**Fig. 1:**
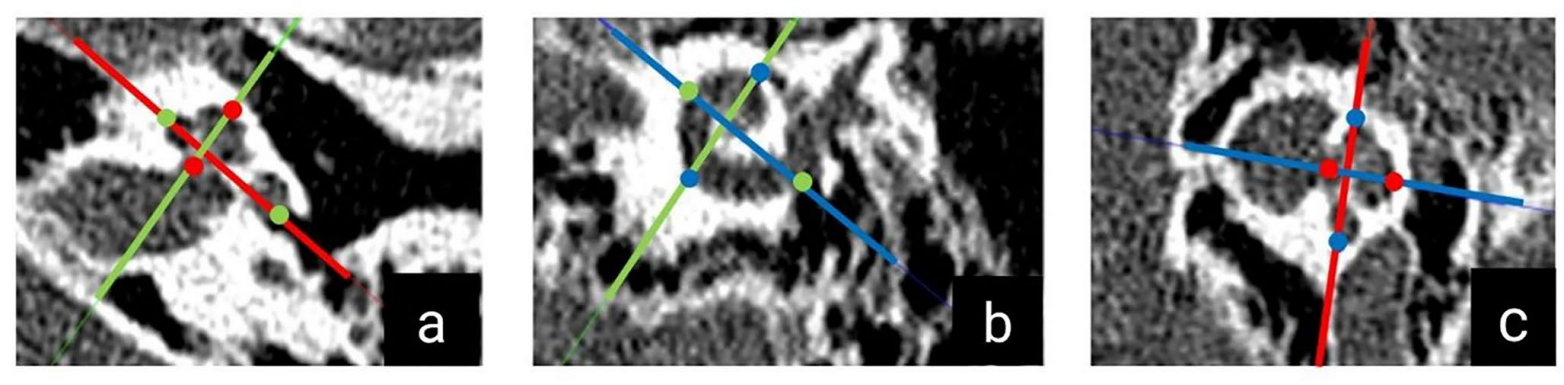
CT images in the OTOPLAN visualisation. The coloured markings were placed by the evaluator and show the diameter (green dot on red line / A value), the width (blue dot on red line / B value) and the height (red dot on green line / H value) of the cochlea. Axial, coronal and sagittal CT views are shown respectively in **a**, **b**, and **c**

The preoperative and postoperative radiological images (139 images each) were measured using OTOPLAN (version 3). For this purpose, the DICOM data sets of the preoperative CT were transferred to the software. A CI surgeon trained and experienced in the use of OTOPLAN then marked all the necessary landmarks and performed all measurements. In the ‘cochlear overview’ generated by the software, the parameters diameter (A), width (B) and height (H) were measured after the landmarks had been placed (Fig. 1). The program used the stored ‘Elliptic Circular Approximation’ algorithm to calculate the length of the cochlear duct (CDL). The simulation of the electrode array implanted in each patient was then performed, taking measurements for all twelve electrode contacts (#1 apical to #12 basal). The insertion angle was given in degrees (°), the insertion depth in millimetres (mm) and the frequency in Hertz (Hz).

The same procedure was used for the postoperative CBCT data sets. In addition, the OTOPLAN program automatically detects the position of the inserted electrode array, which generates the insertion depth, insertion angle and frequency assignment for the individual electrode contacts of the implanted electrode.

### Statistical analysis

All analyses were conducted using the software JASP. For statistical analysis, cochlear measurements obtained from CT and CBCT were compared to assess significant differences. In addition, CT measurements for male and female ears were reviewed and compared within this study. The post-operative electrode insertion values recorded by CBCT were compared with the preoperative values predicted by CT. The groups of measurements were first tested for normal distribution using the Shapiro-Wilk test. For groups with normally distributed values, the difference between paired values was tested for significance using the Student’s t-test; for groups with non-normally distributed values, the Wilcoxon test was used. A p-value of less than 0.05 was considered significant. Outliers in the figures were defined as values falling outside the 95% confidence interval. The results of the cochlear parameters were presented as box plots and the electrode contacts were presented as a scatter plot. The median was used for evaluation.

## Results

### Cochlear measurements

**Fig. 2:**
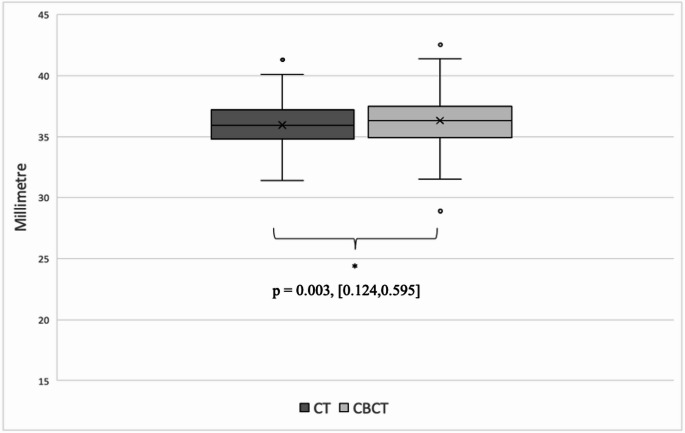
Boxplot of cochlear duct length (CDL) calculations with OTOPLAN: The box plot shows the calculated CDL for the 139 data sets of the CT group (dark grey) and the CBCT group (light grey) in millimetres. A significant difference between the two groups has been statistically proven

For A, there was a normal distribution of values obtained preoperatively by CT in the entire collective. In contrast, the CBCT values were not normally distributed. The median CT value was 9.2 mm (SD 0.41; min. 8.3 mm, max. 10.4 mm), whereas the median CBCT value was 9.5 mm (SD 0.62; min. 7.3 mm, max. 10.9 mm). The Wilcoxon signed rank test showed a significant difference between CT and CBCT values (*p* = < 0.001). Values for H were normally distributed for both measurements. The median value for CT was 4.2 mm (SD 0.36; min. 3.4 mm, max. 5.0 mm) and 4.3 mm (SD 0.42; min. 3.2 mm, max. 5.6 mm) for CBCT measurements. The difference between groups was considered significant by t-test (*p* = < 0.001). For B, normally distributed values were obtained for the CT measurement, whereas the CBCT values did not show a normal distribution. The median value for both CT and CBCT was 6.8 mm (CT SD 0.38; min. 5.7 mm, max. 7.9 mm; CBCT SD 0.44; min. 5.3 mm, max. 8.1 mm). The Wilcoxon signed rank test showed no significant difference between the two groups (*p* = 0.171). The CDL calculated from the above parameters showed a normal distribution in both preoperative CT and postoperative CBCT. A t-test showed a significant difference between the groups (*p* = 0.003). The median value for CT was 35.9 mm (SD 1.74; min. 31.4 mm, max. 41.3 mm), while the median value for CBCT was 36.3 mm (SD 2.01; min. 28.9 mm, max. 42.5 mm) (Fig. 2).

### CT measurements of the cochlea in women and men

**Fig. 3:**
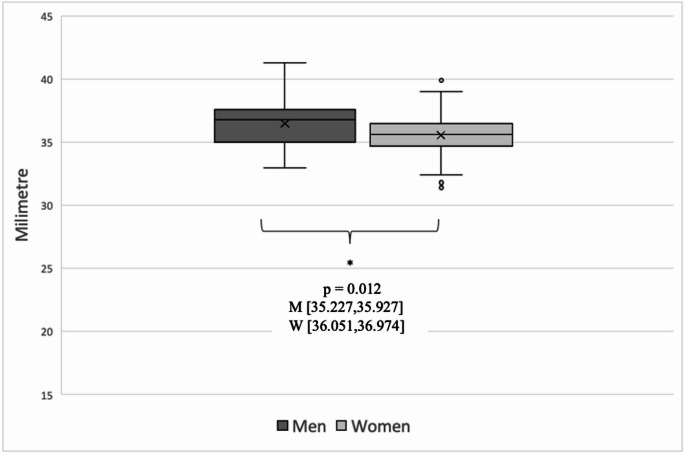
Boxplot of cochlear duct length (CDL) calculated with OTOPLAN for men and women The box plot shows the CDL for the 139 preoperative CT scans, divided into male (dark grey) and female (light grey) groups. The significant difference in cochlear size between the sexes is evident, with CDL being significantly shorter in females than in males

Of the 139 CI ears included in the study, 84 (60%) were female and 55 (40%) were male. Analysis of the CT measurement groups (A, B, H and CDL) showed a normal distribution for both sexes. The median values for A were 9.1 mm (SD 0.40; min. 8.3 mm, max. 10.4 mm) for females and 9.4 mm (SD 0.39; min. 8.6 mm, max. 10.2 mm) for males. A was significantly smaller in women than in men, with a difference of 0.3 mm (*p* = 0.007). For B, the median values were 6.7 mm (SD 0.35; min. 5.7 mm, max. 7.5 mm) in females and 6.9 mm (SD 0.39; min. 6.2 mm, max. 7.9 mm) in males, with a difference of 0.2 mm. Again, B was significantly smaller in females than in males (*p* = 0.046). H showed consistent median values of 4.2 mm in both sexes (women: SD 0.35; min. 3.4 mm, max. 5.0 mm; men: SD 0.36; min. 3.4 mm, max. 5.0 mm), so there was no significant difference in cochlear height between females and males (*p* = 0.143). The median CDL was 35.6 mm (SD 1.64; min. 31.4 mm, max. 39.9 mm) in women and 36.8 mm (SD 1.75; min. 33.0 mm, max. 41.3 mm) in men. This difference was statistically significant (*p* = 0.012, Fig. 3), with females having a shorter CDL.

### Single electrode contact insertion angles

The CT images showed a normal distribution of insertion angles for contacts 2, 3 and 4, whereas the values for contact 1 and contacts 5 to 12 were not normally distributed. In the CBCT images, the values for contacts 2 to 4, 7 and 12 showed a normal distribution; there were no normally distributed values for contacts 1, 5, 6 and 8 to 11.

**Fig. 4:**
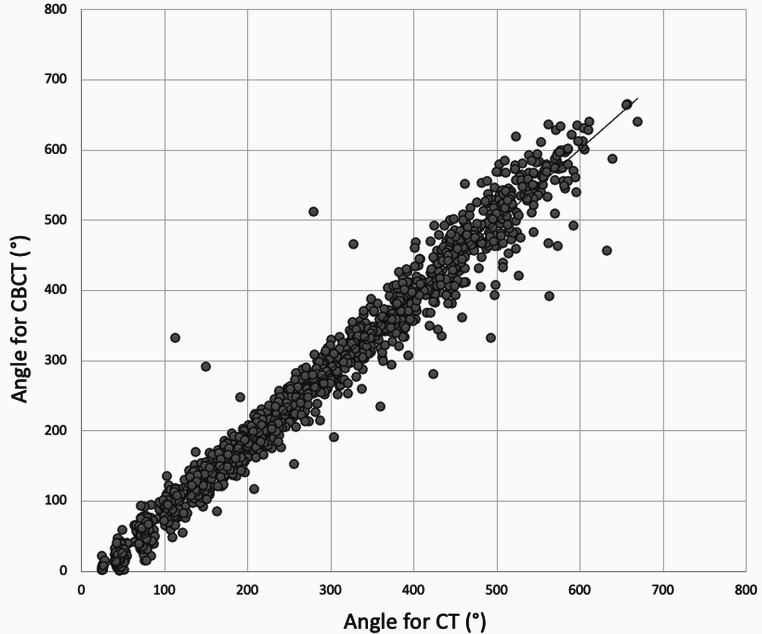
Scatter plot of the insertion angles of the 12 electrode contacts of all 139 cochleae measured in CT (x-axis) and CBCT (y-axis). Electrode contact 1 is most apical (high angle), contact 12 most basal (low angle)

Comparison of insertion angles between CT and CBCT showed the following results in degrees, including calculation of significance (Table 1; Fig. 4):

**Table 1:** Comparison of insertion angles between CT and CBCT

Electrode contact	Median value of insertion angle difference (°)	*P* value	Standard deviation	Minimum (°)	Maximum (°)
1	**9.9**	**p = 0.002**	37.72	-175.2	96.6
2	-0.1	*p* = 0.729	35.70	-170.4	90.9
3	**-7.8**	**p = 0.002**	31.44	-159.9	67.3
4	**-12.9**	**p = < 0.001**	25.25	-142.9	44.5
5	**-11,6**	**p = < 0.001**	24,70	-124,8	138,7
6	**-13,8**	**p = < 0.001**	28,42	-113,5	233,2
7	**-16,6**	**p = < 0.001**	17,43	-102,4	18,6
8	**-16,1**	**p = < 0.001**	17,25	-90,5	57,5
9	**-17,4**	**p = < 0.001**	20,56	-77,2	141,3
10	**-17,6**	**p = < 0.001**	25,08	-66,5	219,6
11	**-21,6**	**p = < 0.001**	14,07	-62,8	21,6
12	**-24,6**	**p = < 0.001**	9,58	-48,4	2,5

The significant differences are marked in bold

The results show significant differences in insertion angles between CT and CBCT, suggesting structural differences in the measurement and imaging of the cochlea.

It is noteworthy that some of the predicted insertion angles in the preoperative CT scan were significantly higher than the actual angles measured in the postoperative CBCT scan. The data in Fig. 4 shows some extreme outliers, suggesting that, for a few patients, the electrodes may have been inserted deeper or shallower than initially predicted.

### Insertion depth

In CT imaging, the measured values for insertion depth in millimetres were not normally distributed for all electrode contacts. Similarly, CBCT measurements were not normally distributed for contacts 1 to 11, but were normally distributed for contact 12.

The difference in insertion depths in millimetres between CT and CBCT was investigated using the Wilcoxon test, with the following results for each contact in terms of median values and significance (Table 2):

**Table 2:** Comparison of insertion depth between CT and CBCT

Electrode contact	Median value of insertion depth difference (mm)	*P* value	Standard deviation	Minimum (mm)	Maximum (mm)
1	**0.6**	**p = < 0.001**	1.35	-7.5	4.0
2	**0.3**	**p = 0.006**	1.24	-6.3	3.3
3	-0.1	*p* = 0.706	1.12	-5.0	42.7
4	**-0.3**	**p = < 0.001**	1.00	-4.7	2.2
5	**-0.3**	**p = < 0.001**	1.01	-4.6	4,7
6	**-0.5**	**p = < 0.001**	1.17	-4.5	8,2
7	**-0.7**	**p = < 0.001**	0,87	-4.3	1,4
8	**-0.8**	**p = < 0.001**	0,88	-4.1	2,7
9	**-0.9**	**p = < 0.001**	1,07	-3,8	6,6
10	**-1,0**	**p = < 0.001**	1,32	-3,8	10,3
11	**-1,4**	**p = < 0.001**	0,97	-4,3	1,4
12	**-1,8**	**p = < 0.001**	0,73	-3,5	0,2

The significant differences are marked in bold

These results highlight significant differences in insertion distance between CT and CBCT measurements, particularly for contacts 1, 2, 4 and 5 to 12.

### Calculation of assigned acoustic frequencies

In CT imaging, the calculated frequency values in Hertz at all electrode contacts did not show a normal distribution. In CBCT imaging, the values for contacts 1 to 11 also showed no normal distribution, whereas contact 12 was normally distributed. The difference in frequency assignment between CT and CBCT values was analysed using the Wilcoxon test, with the following results for each contact (Table 3):

**Table 3:** Comparison of frequency between CT and CBCT

Electrode contact	Median value of frequency difference (Hz)	*P* value	Standard deviation	Minimum (Hz)	Maximum (Hz)
1	**-21.6**	**p = 0.006**	83.24	-180.2	363.8
2	1.9	*p* = 0.807	105.66	-222.0	477.9
3	**29,9**	**p = < 0.001**	-232,9	-232,9	645,0
4	**67,4**	**p = < 0.001**	-243,6	243,6	873,3
5	**101,8**	**p = < 0.001**	-614,6	-614,6	1140,7
6	**167,4**	**p = < 0.001**	-1146,2	-1146,2	1479,6
7	**282,5**	**p = < 0.001**	-357,3	-357,3	1830,5
8	**363,8**	**p = < 0.001**	-902,1	-902,1	2275,8
9	**539,6**	**p = < 0.001**	-2326,1	-2326,1	2802,8
10	**824,2**	**p = < 0.001**	-3908,6	-3908,6	3777,3
11	**1566,8**	**p = < 0.001**	-1241,4	-1241,4	5737,1
12	**2690,6**	**p = < 0.001**	-251,1	-251,1	5737,3

The significant differences are marked in bold

These results show significant differences in frequency assignments between CT and CBCT, especially for contacts 1, 3–12.

## Discussion

Today, various imaging techniques are used to measure the cochlea [22]. Koch et al. showed in their review that the measurement methods for calculating the CDL have developed and improved over decades [23]. Today, the most accurate method for assessing intracochlear structures is micro-CT imaging or histology. However, as these methods are not clinically feasible, CT-based three-dimensional reconstruction of the cochlea is the most reliable imaging method for CDL measurement to date. In this study, a comparison was made between the imaging modalities routinely used in the investigating clinic, namely CT and CBCT. Although comparisons between other imaging modalities have been documented in the literature, such as the study by Müller-Graff et al. [24], to the authors’ knowledge no study has explicitly compared CT and CBCT for cochlear measurement.

Parouris et al. compared the manual measurement of cochlear parameters with the automatic measurement method of OTOPLAN in 109 ears (56 patients) in 2023. The study evaluated inter-rater reliability and performance time for manual and automated results. Analysis included A, B, H-value and CDL. Excellent inter-rater reliability, high correlation of results and reduced time to perform were observed with the automated method [25]. Michael W Canfarotta et al. also analysed the reliability of OTOPLAN in determining the insertion depth, CDL and frequency of individual electrodes in CI recipients in their study. The intraclass correlation coefficients for intra- and interrater reliability of insertion depth of the apicalmost electrode contact, CDL and frequency were excellent. Thus, this study demonstrated excellent interrater agreement in the determination of insertion depth, CDL and corresponding frequency [26]. In this study, interrater discussion was not used as an experienced surgeon performed all measurements.

Rader et al. demonstrated that pitch matching in CI users with single-sided deafness revealed that place-dependent stimulation rates enable a level of tonotopic pitch perception restoration previously unmatched [27]. This suggests that the estimations provided by OTOPLAN could be validated through pitch perception tasks in these patients. It is expected that sound processing strategies incorporating place-dependent stimulation rates will improve pitch perception in CI users.

### Comparison of different imaging modalities

In their retrospective study, Avallone et al. [28] evaluated the accuracy of OTOPLAN predictions using CT images. Most of the prediction errors of the analysed cases remained below 45°. In general, the prediction errors increased from the base to the apex and were greater for longer electrode arrays. Hardly any significant differences were found between the two prediction methods (original version based on a formula proposed by Escudé et al. vs. new version, the so-called elliptical-circular approximation). As a result, the colleagues concluded that the new version of the platform, equipped with an updated prediction module, enables reliable estimation of insertion angles based on CT images, even for cochleae with diverse anatomical variations. Similar to our findings, they observed increasing variability in the predicted cochlear parameters along the electrode array. However, in their case, the variability increased from the base toward the apex, whereas our results revealed the opposite trend. Despite this notable discrepancy, the clinical relevance appears minimal and is likely attributable to differences in imaging protocols and computational methods.

The study by Taeger et al. [29] compared the length of the cochlear duct on CT and magnetic resonance imaging (MRI) in 42 patients. As no clinically relevant differences were found between the two imaging modalities, the concept of radiation-free imaging prior to cochlear implantation was proposed. Weber et al. [30] also compared CT and MRI measurements in 10 patients, corresponding to 20 cochleae, and compared the CDL measurements of both imaging modalities. They found a slightly longer cochlea on MRI, with a small and probably clinically irrelevant difference of 0.89 mm. The same study showed that there was no inter-rater difference between the measurements of experienced CI surgeons and those of less experienced surgeons. Similarly, the study by George-Jones et al. [31] compared the intra- and interobserver variability of CDL measurements using preoperative MRI versus CT using the OTOPLAN planning software in 21 patients. The results showed that the validated otologic-surgical planning software OTOPLAN provides comparable performance to the gold standard CT in the preoperative estimation of CDL using MRI images.

A comparison of CT and X-ray images is also available in the literature. The study by Yoshimura et al. [32] compared preoperative measurements of CDL and insertion angle using OTOPLAN software with postoperative measurements after lead insertion without OTOPLAN using radiographs in 105 patients. There was no significant difference in CDL between the two measurement methods.

Our study showed that there was a small but significant difference between the measured values generated by OTOPLAN between CT and CBCT results. For example, the measurement of the cochlea already showed a difference for the parameters A, H and CDL; only for B no significantly different results could be obtained between the two imaging methods. The data values for insertion angle, insertion depth and frequency generated from the CT data set differed significantly from the postoperative CBCT calculations for the majority of contacts. The difference between CT and CBCT increased for insertion angle, insertion depth and assigned frequency. The smallest difference was found in the apical region of the cochlea. With a mean (median) difference of 21.6 Hz (~ 6%) in the apical region, the frequency difference is approximately the musical interval of a minor second. At the most basal electrode, the difference is around 30% (equivalent to the musical interval of a fourth). The absolute mean difference of 2685 Hz is approximately in the range of the bandwidth of a critical band. Furthermore, the frequency range of the most basal electrode is outside of the frequency range currently used by the manufacturers. Therefore, both apically and basally, at least with the currently used speech coding strategies and the spread of the electric field, clinical relevance cannot be assumed.

In conclusion, there were differences between the preoperative CT and postoperative CBCT measurements. The median differences were 0.2 mm for A, 0.1 mm for H, 0 mm for B and 0.2 mm for the CDL. Although these differences are statistically significant, the magnitude of the values suggests that they are unlikely to be clinically relevant in routine clinical practice. In light of these findings, it can be concluded that our study, which included a larger number of patients than the Chinese study by Yoshimura et al. [32] and the Slovakian study by Paouris et al. [25], confirms that preoperative CDL measurement using the OTOPLAN software has a high degree of predictive accuracy, with a significant difference of 0.2 mm. This supports the proposition that this measurement tool should be used in daily clinical practice to select the optimal individual electrode array length for each case. Furthermore, this method has the advantage that it can be performed by the ENT specialist in a relatively short time. An intraoperative image could potentially improve image quality, as patient movement is minimised during surgery, making it advantageous to acquire an image intraoperatively if available.

### Gender-specific size of the cochlea

The present study showed that the cochlea is significantly larger in males than in females. CT-based calculations of the length of the cochlear duct were 36.8 ± 1.7 mm in males (CBCT: 36.5 ± 0.2 mm) and 35.6 ± 1.6 mm in females (CBCT: 35.8 ± 0.3 mm). Spiegel et al. [16] also showed that the cochlea of men is significantly larger than that of women. It can therefore be postulated that women would benefit from shorter electrode arrays for individualised treatment. In cases where OTOPLAN cannot be used to select the appropriate electrode preoperatively, it is not advisable to recommend the use of the longest available electrode as the standard option, given the goal of preserving structure. Similarly, Baguant et al. [33] compared the length of the CDL between male and female patients in 880 CTs performed between 2014 and 2020. The results showed that the mean CDL was 34.5 mm in the male cohort and 33.3 mm in the female cohort. In line with our own findings, the authors concluded that there is a statistically significant difference in CDL between the sexes, with males having a longer CDL compared to females.

### Clinical implications for patients with a malformed cochleae

Eisenman et al. demonstrated that children with cochlear malformations can benefit from cochlear implantation using multichannel devices, and may ultimately achieve outcomes comparable to those of children with anatomically normal cochleae [34]. Furthermore, Kocabay et al. reported that pediatric CI users with inner ear malformations require individualized adjustments to fitting parameters [35]. These findings suggest that personalized, preoperative cochlear measurements could enhance hearing outcomes in this patient population.

### Limitations of the study

When CT and CBCT measurements were compared, the Shapiro–Wilk test revealed that the CBCT-derived values were not normally distributed. This suggests that the data were skewed or contained outliers, possibly due to factors such as image resolution, reconstruction algorithms or inherent variability in CBCT measurement.

It should be noted that the present study used two different imaging techniques, namely CT and CBCT, to record pre- and post-operative values. CBCT imaging is significantly more susceptible to the introduction of motion artefacts, which can result in a blurred image. This may be the reason for the significant discrepancies between CT and CBCT measurements. Although a postoperative CT scan could be performed, it is preferable to avoid this option due to the increased radiation exposure. According to the recommendations of Müller-Graff et al. [22], the aim should be to perform preoperative imaging with the highest possible resolution to ensure the accuracy of the software measurement and thus provide a good predictive value. However, for post-operative determination of the electrode array position, the use of CBCT is an acceptable alternative as the radiation exposure is low and the accuracy is still sufficient.

Moreover, OTOPLAN is a software tool developed and approved exclusively for use with MED-EL electrode carriers. Consequently, it is not compatible with CIs from other manufacturers. Therefore, our results cannot be generalised to all types of CI.

In addition, minor variations in the inter-rater reliability of cochlear measurements may be evident. The impact of inter-rater variability was not assessed due to the sole involvement of a single rater in the evaluation process.

## Conclusion

In conclusion, the OTOPLAN software was shown to be an effective tool for predicting the insertion depth of CI electrode arrays with a high degree of accuracy. When using the software, a statistically significant difference between CT and CBCT measurements of the cochlea was observed. However, the magnitude of this difference is not considered to be clinically significant. It is therefore recommended that high resolution imaging is obtained preoperatively to select the optimal electrode array length. In the post-operative period, CBCT is sufficiently reliable to allow precise control of the position of the electrode array with a minimum of radiation exposure.

Female cochlea size was found to be statistically significantly smaller than that of male cochlea. In the absence of preoperative measurements, this information can be used by the surgeon to guide the selection of a sex-specific standard electrode.

## Data Availability

Upon reasonable request, pseudonymised data from this study are available from the authors.
